# Redundancy Removal Adversarial Active Learning Based on Norm Online Uncertainty Indicator

**DOI:** 10.1155/2021/4752568

**Published:** 2021-10-25

**Authors:** Jifeng Guo, Zhiqi Pang, Wenbo Sun, Shi Li, Yu Chen

**Affiliations:** College of Information, Northeast Forestry University, Harbin 150040, China

## Abstract

Active learning aims to select the most valuable unlabelled samples for annotation. In this paper, we propose a redundancy removal adversarial active learning (RRAAL) method based on norm online uncertainty indicator, which selects samples based on their distribution, uncertainty, and redundancy. RRAAL includes a representation generator, state discriminator, and redundancy removal module (RRM). The purpose of the representation generator is to learn the feature representation of a sample, and the state discriminator predicts the state of the feature vector after concatenation. We added a sample discriminator to the representation generator to improve the representation learning ability of the generator and designed a norm online uncertainty indicator (Norm-OUI) to provide a more accurate uncertainty score for the state discriminator. In addition, we designed an RRM based on a greedy algorithm to reduce the number of redundant samples in the labelled pool. The experimental results on four datasets show that the state discriminator, Norm-OUI, and RRM can improve the performance of RRAAL, and RRAAL outperforms the previous state-of-the-art active learning methods.

## 1. Introduction

In recent years, image processing tasks based on deep learning [1–3] have achieved great success, but they mainly rely on a large number of labelled datasets. Although supervised learning has better performance than semisupervised learning [4–10] and self-supervised learning [11], it is highly dependent on labelled data. In reality, it is very difficult or even unrealistic to obtain a large number of labelled datasets in many fields, and it inevitably consumes many resources [12, 13]. To mitigate the impact of such problems, some researchers have proposed active learning [14, 15]. The process of active learning is to select or synthesize the most useful samples from the unlabelled samples for model training, then use Oracle to label the selected samples, and finally add the labelled samples to the labelled pool to update the task model for training. The process is repeated until the performance of the task model meets the requirements or the label budget is exhausted. At present, active learning has been widely used in image classification [16–18] and segmentation [19, 20] tasks and has made some achievements.

The recently proposed SRAAL method [21] uses annotated information and labelled/unlabelled state information to select samples and has achieved competitive performance. SRAAL inherited VAAL's idea of adversarial learning [22]; that is, SRAAL uses the adversarial approach [23, 24] to learn the feature representation of labelled samples and unlabelled samples and selects samples that can increase the diversity of the labelled pool according to the distribution of samples. In addition, SRAAL sets up an online uncertainty indicator (OUI) for the unlabelled samples to calculate the contribution of this sample to the model. The OUI considers the influence of the maximum element and variance in the category vector on the uncertainty. In summary, SRAAL comprehensively considers the diversity and uncertainty of samples.

We performed a visual analysis of the samples selected by SRAAL [21] and found that some samples have extremely high similarity. In this paper, we refer to similar samples as redundant samples. Redundant samples increase the annotation cost but contribute little to improvements in model performance. To solve this problem, we designed the redundancy removal module (RRM), which defines the threshold value for the feature distance of samples, effectively avoiding the influence of redundant samples.

In addition, we found in the experiment that the OUI used the whole category vector to calculate the uncertainty score, and the score was positively correlated with the variance of the vector. This is not a good option. The reasons are as follows: due to the introduction of softmax, most elements in the category vector of the sample are close to zero, and we call these elements “tiny values.” For a dataset with a small number of categories, these tiny values can seriously affect the variance. For example, for a dataset with 10 categories, the variances of the vectors [0.6, 0.4, 0,0,0,0,0,0,0,0] and [0.5, 0.5, 0,0,0,0,0,0,0,0] are 0.42 and 0.40, respectively, which are very similar numerically, while in fact, their uncertainties are quite different. To remedy this drawback, we designed a new OUI named Norm-OUI, which no longer relies on the variance to calculate the uncertainty but uses the *p*-norm of the vectors. Norm-OUI is more sensitive to the uncertainty of vectors.

The main contributions of this paper are summarized as follows:

1. We propose a redundancy removal adversarial active learning (RRAAL) method based on norm online uncertainty indicator, which fully considers the diversity, uncertainty, and redundancy of samples
2. We design a sample discriminator to improve the representational learning ability of the generator and proposed a Norm-OUI based on the *p*-norm to calculate the uncertainty score of the samples
3. We design an RRM to remove redundant samples and thus reduce inefficient labelling

## 2. Related Work

Current mainstream active learning methods can be divided into synthesis-based methods [25, 26] and pool-based methods [27–29]. The method in this paper is a pool-based method. Pool-based methods can be divided into uncertainty-based and distribution-based methods.

Uncertainty-based methods [30–33] select the most uncertain samples for the model in each iteration. For example, in the realm of Bayesian frameworks, Gaussian processes [30, 31] are used to assess the uncertainty of samples. In addition, Bayesian optimization [34, 35] has many application scenarios. In the realm of non-Bayesian frameworks, the distance from the decision boundary [32] and expected risk minimization [33] are used to assess the uncertainty of samples. Yoo et al. proposed a method based on a loss prediction module (LPM) [36] to predict the sample uncertainty. Uncertainty-based methods often depend on the performance of the task model, and the samples selected are directly related to the task model.

Distribution-based methods [22, 37] tend to select samples that increase the diversity of the labelled pool. By taking advantage of the image distance, a core-set approach [37] can select a set of data points from an unlabelled dataset and obtain a result that a model learned from the selected subset that is competitive for the remaining data points. VAAL [22] uses the adversarial learning [23, 24] of a variational autoencoder (VAE) [38] and discriminator to learn the feature representations of labelled samples and unlabelled samples and then uses the difference between them to make a sample selection. In essence, the method selects samples based on their diversity, which is not equal to the amount of information contained in a sample, so the results of the method may be unreliable. SRAAL [21] uses annotated information and labelled/unlabelled state information to select samples and fully considers the distribution and uncertainty of the samples. Our method also takes into account the uncertainty and diversity of the samples. In addition, we also consider the redundancy of the samples. The experimental results verify that RRAAL is superior to the existing pool-based methods.

The purpose of the synthesis-based methods is to synthesize the most useful samples for the model by using the generated model [24, 39]. The idea was first proposed in GAAL [25], which uses a GAN to generate samples closer to the decision boundary than the existing samples. BGADL [26] combines BDA [40] and BALD [41] to perform iterative training on the task model and the generated model, thus improving the performance of the task model. Similarly, ARAL [42] also uses generated images to update the task model. Synthesis-based methods have higher complexity, and their performance depends on the performance of both the generation model and the task model.

## 3. Method

**Figure 1:**
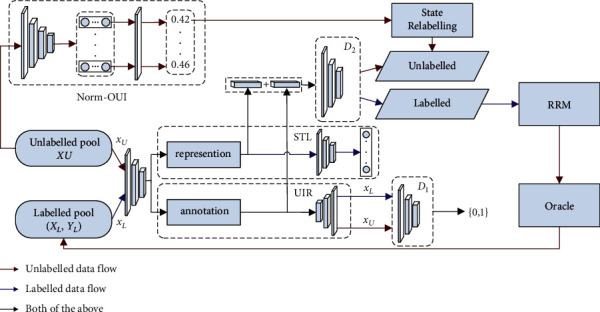
Network architecture of RRAAL. *X*_*U*_ represents the unlabelled pool, and (*X*_*L*_, *Y*_*L*_) represents the labelled pool, where *Y*_*L*_ is the label set. We used Oracle to label the unlabelled samples.

In this section, we describe the RRAAL model presented in this paper. RRAAL selects the unlabelled sample with the most information based on the uncertainty, distribution, and redundancy of the sample, and its overall architecture is shown in Figure 1. RRAAL is composed of a representation generator (Section 3.2), a state discriminator (Section 3.3), and an RRM (Section 3.4). The representation generator is used to learn the feature representations of both labelled and unlabelled samples. The state discriminator predicts the state value of the sample according to the concatenated feature vector. The RRM selects a set of samples with the lowest redundancy on this basis. Section 3.4 introduces the sampling strategy based on the above three modules.

### 3.1. Unified Representation Generator

The unified representation generator of SRAAL includes an encoder, an unsupervised image reconstructor (UIR), and a supervised target learner (STL). The UIR learns the feature representation of the sample by reconstructing the sample, while the STL is used to embed the annotation information of the sample into the representation. The UIR is composed of transposed convolutional layers, and the STL is similar in structure to the task model. To improve the reconstruction ability of the UIR and further improve the ability of the encoder and UIR to learn the sample representation, we added a sample discriminator *D*_*1*_ after the UIR to guide the reconstruction process of the encoder and UIR, as shown in Figure 1. The optimization objective of the sample discriminant *D*_*1*_ is defined as follows:

(1) $$\begin{array}{c} L_{D_{1}}=L_{D_{1}}^{U}+L_{D_{1}}^{L},\\ L_{D_{1}}^{U}=-E\left[\log\;D_{1}\left(x_{U}\right)\right]-E\left[\log\left(1-D_{1}\right)\left(x_{U}^{\prime }\right)\right],\\ L_{D_{1}}^{L}=-E\left[\log\;D_{1}\left(x_{L}\right)\right]-E\left[\log\left(1-D_{1}\right)\left(x_{L}^{\prime }\right)\right], \end{array}$$

where *x*_*L*_ and *x*_*U*_ are real labelled samples and unlabelled samples, respectively, while *x*_*L*_′ and *x*_*U*_′ are the generated labelled samples and unlabelled samples, respectively.

The optimization objective of the UIR is defined as follows:

(2) $$\begin{array}{c} L_{UIR}=L_{UIR}^{U}+L_{UIR}^{L},\\ L_{UIR}^{U}=E\left[\log\left[p_{\phi }\left(x_{U}|z_{U}\right)\right]-D_{KL}\left(q_{\theta }\left.\left(z_{U}|x_{U}\right)\right\|p\left(z\right)\right)\right]-L_{D_{1}}^{U},\\ L_{UIR}^{L}=E\left[\log\left[p_{\phi }\left(x_{L}|z_{L}\right)\right]-D_{KL}\left(q_{\theta }\left.\left(z_{L}|x_{L}\right)\right\|p\left(z\right)\right)\right]-L_{D_{1}}^{L}, \end{array}$$

where *L*_UIR_^*U*^ is the objective function of the unlabelled sample, *L*_UIR_^*L*^ is the objective function of the labelled sample, *z* is the feature representation, *ϕ* parametrizes the decoder *p*_*ϕ*_, and *θ* parametrizes the encoder *q*_*θ*_.

Finally, the UIR reconstructs the image under the guidance of *D*_*l*_ and learns the feature representation of the sample. Previous experiments have indicated that adding annotation information can improve the performance of active learning models [29]. We use the same STL as in SRAAL, whose objective function is defined as follows:

(3) $$\begin{array}{c} L_{\text{STL}}=E\left[\log\left[p_{\phi }\left(y_{L}|z_{L}\right)\right]-D_{KL}\left(\left.q_{\theta }\left(y_{L}|x_{L}\right)\right\|p\left(z\right)\right)\right]. \end{array}$$

Because of the dependency on the label of the sample, the STL can only be trained using the labelled sample. Finally, the feature representation learned by the UIR and the annotation information learned by the STL are concatenated as the final sample representation.

### 3.2. State Discriminator and State Relabelling

Considering that the uncertainty score calculated by the OUI will be affected by a tiny value in the category vector, we designed a new indicator function: the Norm-OUI. In this method, the variance of the vector is no longer used to calculate the uncertainty score, but the *p*-norm of the vector is calculated. Therefore, we redefined the uncertainty score function as follows:

(4) $$\begin{array}{c} \text{score}\left(x_{U}\right)=1-\frac{\min\text{ norm}\left(V\right)}{\text{norm}\left(V\right)}\times \max\left(V\right), \end{array}$$

where *V* is the category vector and max(*V*) is the largest element in vector *V*. Mi*n* norm(*V*) is defined as follows:

(5) $$\begin{array}{c} \min\text{ norm}\left(V\right)={\left[{\left(\max\left(V\right)\right)}^{p}+\left(C-1\right){\left(\frac{1-\max\left(V\right)}{1-C}\right)}^{p}\right]}^{1/p}. \end{array}$$

By definition, min norm(*V*) is the minimum *p*-norm for all the vectors whose largest element is max(*V*). The objective function of the state discriminator *D*_2_ is as follows:

(6) $$\begin{array}{c} L_{D}=-E\left[\log\left(D_{2}\left(q_{\theta }\left(z_{L}|x_{L}\right)\right)\right)\right]-E\left[\log\left(\text{score}\left(x_{U}\right)-D_{2}\left(q_{\theta }\left(z_{L}|x_{L}\right)\right)\right)\right], \end{array}$$

where score(*x*_*U*_) is the new state value of unlabelled sample *x*_*U*_. The objective function of the representation generator in the adversarial learning process with *D*_2_ is as follows:

(7) $$\begin{array}{c} L_{\text{adv}}=-E\left[\log\left(D_{2}\left(q_{\theta }\left(z_{L}|x_{L}\right)\right)\right)\right]-E\left[\log\left(D_{2}\left(q_{\theta }\left(z_{U}|x_{U}\right)\right)\right)\right]. \end{array}$$

The total objective function for the representation generator is as follows:

(8) $$\begin{array}{c} L_{G}=\lambda_{1}L_{\text{UIR}}+\lambda_{2}L_{\text{STL}}+\lambda_{3}L_{\text{adv}}, \end{array}$$

where *λ*_1_, *λ*_2_, and *λ*_3_ are hyperparameters that control the ratio of the function.

### 3.3. RRM

The purpose of an RRM is to remove redundant samples based on the state value predicted by the state discriminator and the feature distance of the image to reduce the cost of labelling. In this paper, the unlabelled samples are first arranged in descending order according to the predicted values as [*x*_1_, *x*_2_, *x*_3_,…], and then the feature representations learned by the representation generator are normalized. Then, the normalized representations are used to calculate the similarity between samples. The similarity between a pair of samples (*x*_*i*_, *x*_*j*_) is defined as follows:

(9) $$\begin{array}{c} S\left(x_{i},x_{j}\right)=\sqrt{\sum_{k=1}^{n}{\left(N{\left(x_{i}\right)}_{k}-N{\left(x_{j}\right)}_{k}\right)}^{2}}, \end{array}$$

where *N*(*x*_*i*_) and *N*(*x*_*j*_) represent the normalized feature representations of samples *x*_*i*_ and *x*_*j*_, respectively. *N*(*x*_*i*_)_*k*_ and *N*(*x*_*j*_)_*k*_ represent the *k*-th element of the feature representations of *N*(*x*_*i*_) and *N*(*x*_*j*_), respectively. The RRM is based on the greedy algorithm for redundancy removal, and the specific steps are shown in Algorithm 1. The hyperparameter *d* is set in the algorithm to control the feature distance between the two samples, and *X*_*S*_, which is finally returned, is the sample that needs Oracle labelling.

### 3.4. Sampling Strategy in Active Learning

The algorithm for training the RRAAL algorithm at each iteration is shown in Figure 1. In each iteration, the sampling process is divided into two phases. In the first phase, the generator generates feature representations for each sample, and the state discriminator *D*_*2*_ predicts the state value of samples under the guidance of the Norm-OUI. In the second stage, we arrange the unlabelled samples as [*x*_1_, *x*_2_, *x*_3_,…] in descending order according to the predicted value, input this sequence into the RRM for sample selection, and finally obtain the samples that need to be labelled. After each iteration, we need to update the task model and the entire active learning model.

## 4. Experiment

In this section, we evaluate the RRAAL algorithm in both classification and segmentation tasks.

*Dataset*. The datasets we selected in the classification experiment include CIFAR-10 [43], CIFAR-100 [43], and Caltech-101 [44]. Both CIFAR-10 and CIFAR-100 contain 60,000 images, of which 50,000 are training images and 10,000 are test images. CIFAR-10 has 10 categories with 6,000 images per category, while CIFAR-100 has 100 categories with 600 images per category. Caltech-101 contains 101 image categories and a background category, with a total of 9,146 images, with 40 to 800 images per category. The dataset we selected in the segmentation experiment is Cityscapes [45]; its training set contains 2,975 images, the verification set contains 500 images, and the test set contains 1,525 images.

For each dataset, we randomly sampled *M*=10% samples from the entire dataset as the initial labelled pool *X*_*L*_, and the remaining 90% of samples formed the initial unlabelled pool *X*_*U*_. We select *K*=5% samples from the unlabelled pool for labelling each iteration and then position these samples in the labelled pool until the labelled samples reach 40%. For each active learning method, we repeated the experiment five times with a different initially labelled pool and reported the average performance.

*Task Model*. The task model we used in the image classification experiment is ResNet-18 [46], and the task model we used in the segmentation experiment is a DRN [47]. We compared the average accuracy of the task model in the five experiments.

### 4.1. Parameter Analysis

**Figure 2:**
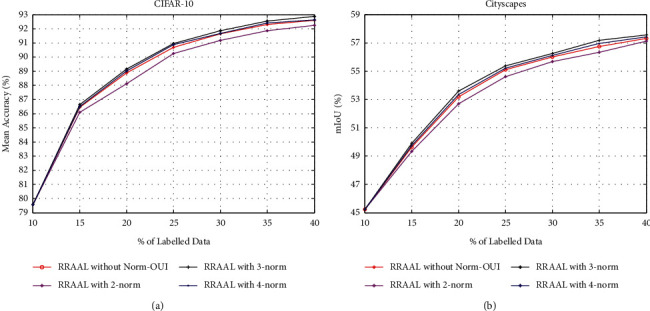
Basic rocket ship design. The rocket ship is propelled with three thrusters and features a single viewing window. The nose cone is detachable upon impact. (a) CIFAR-10. (b) Cityscapes.

To explore the influence of the *p*-norm on the model performance, we conducted a parameter analysis experiment on the CIFAR-10 and Cityscapes datasets. This section compares the model performance with the 2-norm, 3-norm, and 4-norm in the Norm-OUI. To see the effect of the p-norm on the performance more clearly, we added RRAAL without the Norm-OUI (using the OUI) as a reference. The experimental results of the parameter analysis are shown in Figure 2.

As seen from the experimental results, the performance of RRAAL with the 2-norm is the worst on the two datasets and is lower than that of RRAAL without the Norm-OUI. The performance of RRAAL with the 3-norm and RRAAL with the 4-norm is better than that of RRAAL without the Norm-OUI, and RRAAL with the 3-norm achieves the optimal performance. Therefore, the proposed RRAAL finally uses 3-norm.

### 4.2. Ablation Study

To evaluate the contribution of the Norm-OUI, RRM, and sample discriminator *D*_*1*_ introduced in RRAAL, we conducted an ablation study on the CIFAR-10 and Cityscapes datasets. The compared models include RRAAL, RRAAL without the Norm-OUI (using the OUI), RRAAL without the RRM, and RRAAL without both, and SRAAL. It is worth noting that the difference between RRAAL without both and SRAAL is that the former has a sample discriminator *D*_*1*_.

**Figure 3:**
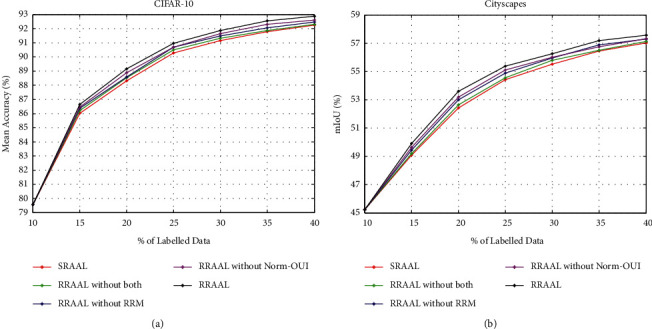
Basic rocket ship design. The rocket ship is propelled with three thrusters and features a single viewing window. The nose cone is detachable upon impact. (a) CIFAR-10. (b) Cityscapes.

Figure 3 shows the results of the ablation study. On the CIFAR-10 and Cityscapes datasets, the experimental results show that the overall performance of RRAAL is always better than that of the other methods, and the performance of RRAAL without both is slightly better than that of SRAAL and lower than that of the other three methods.

The experimental results show that (1) the Norm-OUI, RRM, and *D*_*1*_ can improve the performance of SRAAL; (2) the performance is optimized when the three components are combined.

### 4.3. Classification Experiment

**Figure 4:**
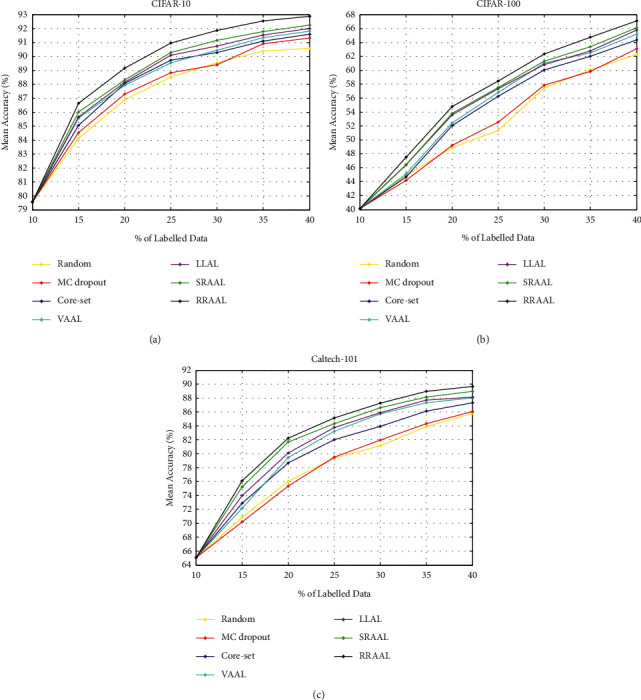
Active learning results of image classification on the (a) CIFAR-10; (b) CIFAR-100; (c) Caltech-101 dataset.

We compare RRAAL with the current mainstream methods: SRAAL [21], LLAL [36], core-set [37], Monte Carlo dropout (MC dropout) [48], VAAL [22], and Random.  Figure 4 shows the experimental results of our proposed RRAAL and other methods on the three datasets. On the CIFAR-10 dataset, RRAAL outperformed the other methods throughout the process. When the data rates were 20%, 30%, and 40%, the mean accuracies of RRAAL were 0.83%, 0.71%, and 0.62%, respectively, higher than those of the second best method (SRAAL). The experimental results show the superiority of RRAAL in datasets with a small number of categories.

The number of categories in the CIFAR-100 dataset is 10 times larger than that in CIFAR-10, which makes the dataset more challenging. On the CIFAR-100 dataset, RRAAL is obviously superior to VAAL, core-set, MC dropout, and Random and slightly superior to SRAAL and LLAL. When the data rates are 20%, 30%, and 40%, the mean accuracies of RRAAL are 0.98%, 1.01%, and 0.98% higher than those of SRAAL and 1.20%, 1.50%, and 1.31% higher than those of LLAL, respectively. Thus, it can be seen that RRAAL still has advantages.

We calculated the final performance of each method on the three datasets, and the results are shown in Table 1. As can be seen from Table 1, compared with other methods, RRAAL achieves the best performance on all three datasets.

In addition, we calculated the computational costs on three datasets. The computational cost of Random is the smallest, so the computational cost of Random is taken as unit 1. The experimental results are shown in Table 2. As can be seen from Table 2, although RRAAL has a higher computational cost, it is very similar to SRAAL and VAAL and achieves higher performance than them. Because the computational cost is much less than the manual cost, RRAAL is still useful.

### 4.4. Segmentation Experiment

**Figure 5:**
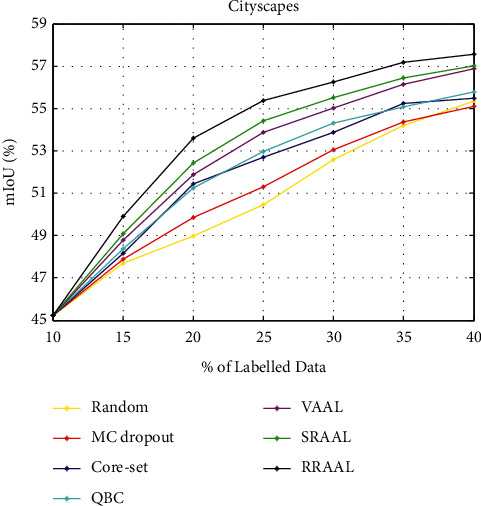
Active learning results of image segmentation on the Cityscapes dataset.

We compare RRAAL with the current mainstream methods: SRAAL [21], VAAL [22], core-set [37], query-by-committee (QBC) [49], MC dropout [48], and Random. Image segmentation is more challenging than image classification.  Figure 5 shows the experimental results of our proposed RRAAL method and the other methods on the Cityscapes dataset. RRAAL has the best performance, and SRAAL and VAAL rank second and third, respectively. The performance of core-set and QBC is similar, which is better than that of MC dropout and Random. When the data rates were 20%, 30%, and 40%, the mIoU of RRAAL was 1.16%, 0.73%, and 0.56 higher than that of SRAAL and 1.70%, 1.23%, and 0.69% higher than that of VAAL, respectively. This result fully verifies the superiority of RRAAL.

## 5. Conclusions

In this paper, we first analysed the problems existing in SRAAL, such as an impractical state indicator function and excessive redundancy, and then proposed RRAAL to solve these problems. RRAAL uses the distribution, uncertainty, and redundancy for sample selection and includes a representation generator, a state discriminator, and an RRM. First, we analysed the parameters of the Norm-OUI and selected the 3-norm. Then, we set up an ablation study to verify the contributions of the Norm-OUI, RRM, and sample discriminator *D*_*1*_. Finally, we verified the effectiveness of RRAAL with classification and segmentation tasks. The performance of RRAAL is 0.62%, 0.98%, and 0.63% higher than that of the state-of-the-art method (SRAAL) in classification datasets and 0.56% higher than that of SRAAL in segmentation datasets. The experimental results show that the overall performance of RRAAL on the four datasets is better than that of the existing mainstream methods.

## Figures and Tables

**Algorithm 1:**
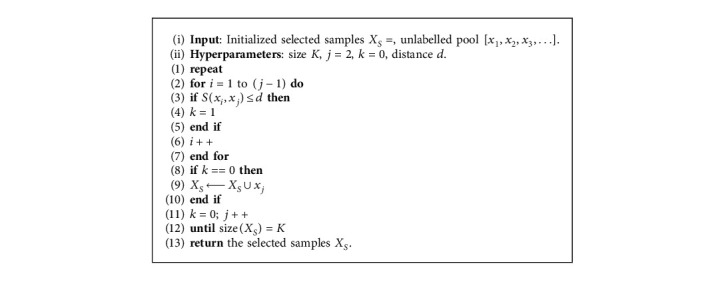
Redundancy removal strategy.

**Table 1:** Experimental results of RRAAL and other methods on CIFAR-10, CIFAR-100, and Caltech-101.

Methods	CIFAR-10	CIFAR-100	Caltech-101
Random	90.59	62.29	85.79
MC dropout	91.33	63.13	86.09
Core-set	91.59	64.39	87.32
VAAL	91.81	65.21	88.06
LLAL	92.01	65.78	88.13
SRAAL	92.25	66.11	88.95
RRAAL	92.87	67.09	89.58

**Table 2:** Time cost of RRAAL and other methods on CIFAR-10, CIFAR-100, and Caltech-101.

Methods	CIFAR-10	CIFAR-100	Caltech-101
Random	1.00	1.00	1.00
MC dropout	1.05	1.06	1.06
Core-set	1.51	1.54	1.52
VAAL	2.07	2.11	2.03
LLAL	1.28	1.31	1.33
SRAAL	2.15	2.18	2.12
RRAAL	2.21	2.17	2.19

## Data Availability

The data used to support this study are available from the corresponding author upon request.

## References

[1] Li C., Guo J., Guo C. (2018). Emerging from water: underwater image color correction based on weakly supervised color transfer. *IEEE Signal Processing Letters*.

[2] Abbott B. P., Abbott R., Abbott T. D. (2016). Observation of gravitational waves from a binary black hole merger. *Physical Review Letters*.

[3] Li C., Guo C., Guo J., Han P., Fu H., Cong R. (2019). PDR-Net: Perception-inspired single image dehazing network with refinement. *IEEE Transactions on Multimedia*.

[4] Kingma D. P., Mohamed S., Rezende D. J., Welling M. (2014). Semi-supervised learning with deep generative models. *Advances in Neural Information Processing Systems*.

[5] Ouali Y., Hudelot C., Tami M. An Overview of Deep Semi-supervised Learning.

[6] Doulamis N., Doulamis A. Semi-supervised deep learning for object tracking and classification.

[7] Lee D. H. Pseudo-label: The simple and efficient semi-supervised learning method for deep neural networks.

[8] Niu G., Jitkrittum W., Dai B., Hachiya H., Sugiyama M. Squared-loss mutual information regularization: a novel information-theoretic approach to semi-supervised learning.

[9] Wang M., Fu W., Hao S., Tao D., Wu X. (2016). Scalable semi-supervised learning by efficient anchor graph regularization. *IEEE Transactions on Knowledge and Data Engineering*.

[10] Protopapadakis E., Doulamis A., Doulamis N., Maltezos E. (2021). Stacked autoencoders driven by semi-supervised learning for building extraction from near infrared remote sensing imagery. *Remote Sensing*.

[11] Zhai X., Oliver A., Kolesnikov A., Beyer L. S4l: self-supervised semi-supervised learning.

[12] Esteva A., Kuprel B., Novoa R. A. (2017). Dermatologist-level classification of skin cancer with deep neural networks. *Nature*.

[13] She Q., Chen K., Luo Z., Nguyen T., Potter T., Zhang Y. (2020). Double-Criteria Active Learning for Multiclass Brain-Computer Interfaces. *Computational intelligence and neuroscience*.

[14] Guo J., Pang Z., Bai M., Xie P., Chen Y. (2021). Dual Generative Adversarial Active Learning. *Applied Intelligence*.

[15] Beluch W. H., Genewein T., Nürnberger A., Köhler J. M. The power of ensembles for active learning in image classification.

[16] Gal Y., Islam R., Ghahramani Z. Deep bayesian active learning with image data.

[17] Sun Y., Zhang J., Zhang Y. Multi-sensor image classification based on active learning.

[18] Tuia D., Ratle F., Pacifici F., Kanevski M. F., Emery W. J. (2009). Active learning methods for remote sensing image classification. *IEEE Transactions on Geoscience and Remote Sensing*.

[19] Jain S. D., Grauman K. Active image segmentation propagation.

[20] Vezhnevets A., Buhmann J. M., Ferrari V. Active learning for semantic segmentation with expected change.

[21] Zhang B., Li L., Yang S., Wang S., Zha Z. J., Huang Q. State-relabeling adversarial active learning.

[22] Sinha S., Ebrahimi S., Darrell T. Variational adversarial active learning.

[23] Goodfellow I., Pouget-Abadie J., Mirza M. Generative adversarial nets.

[24] Guo J., Pang Z., Yang F., Shen J., Zhang J. (2020). Study on the method of fundus image generation based on improved gan. *Mathematical Problems in Engineering*.

[25] Zhu J. J., Bento J. Generative adversarial active learning.

[26] Tran T., Do T. T., Reid I., Carneiro G. Bayesian generative active deep learning.

[27] Wu D. (2018). Pool-based sequential active learning for regression. *IEEE transactions on neural networks and learning systems*.

[28] Geifman Y., El-Yaniv R. (2017). Deep active learning over the long tail. *Computer Science Bibliography*.

[29] Joshi A. J., Porikli F., Papanikolopoulos N. Multi-class active learning for image classification.

[30] Kapoor A., Grauman K., Urtasun R., Darrell T. Active learning with Gaussian processes for object categorization.

[31] Wang H., Gao X., Zhang K., Li J. (2015). Single-image super-resolution using active-sampling Gaussian process regression. *IEEE Transactions on Image Processing*.

[32] Brinker K. Incorporating diversity in active learning with support vector machines.

[33] Wang Z., Ye J. (2015). Querying discriminative and representative samples for batch mode active learning. *ACM Transactions on Knowledge Discovery from Data*.

[34] Chen P., Xia J., Merrick B. M., Brazil T. J. (2016). Multiobjective Bayesian optimization for active load modulation in a broadband 20-W GaN Doherty power amplifier design. *IEEE Transactions on Microwave Theory and Techniques*.

[35] Kaselimi M., Doulamis N., Doulamis A., Voulodimos A., Protopapadakis E. Bayesian-optimized bidirectional LSTM regression model for non-intrusive load monitoring.

[36] Yoo D., Kweon I. S. Learning Loss for Active Learning.

[37] Sener O., Savarese S. Active learning for convolutional neural networks: a core-set approach.

[38] Kingma D. P., Welling M. Auto-encoding variational bayes.

[39] Mirza M., Osindero S. Conditional generative adversarial nets.

[40] Tran T., Pham T., Carneiro G., Palmer L., Reid I. A bayesian data augmentation approach for learning deep models.

[41] Gal Y., Islam R., Ghahramani Z. Deep bayesian active learning with image data.

[42] Mottaghi A., Yeung S. Adversarial representation active learning.

[43] Krizhevsky A., Hinton G. (2009). *Learning Multiple Layers of Features from Tiny Images*.

[44] Fei-Fei L., Fergus R., Perona P. Learning generative visual models from few training examples: an incremental bayesian approach tested on 101 object categories.

[45] Cordts M., Omran M., Ramos S. The cityscapes dataset for semantic urban scene understanding.

[46] Jung H., Choi M. K., Jung J., Lee J.-H., Kwon S., Jung W. Y. ResNet-based vehicle classification and localization in traffic surveillance systems.

[47] Yu F., Koltun V., Funkhouser T. Dilated residual networks.

[48] Gal Y., Ghahramani Z. Dropout as a bayesian approximation: representing model uncertainty in deep learning.

[49] Kuo W., Häne C., Yuh E., Mukherjee P., Malik J. Cost-sensitive active learning for intracranial hemorrhage detection.

